# Catatonia as a debut of systemic lupus erythematosus: a case-report on a diagnostic challenge

**DOI:** 10.1192/j.eurpsy.2025.1221

**Published:** 2025-08-26

**Authors:** H. Andreu, B. Serra-Sarró, L. Bueno, Ò. de Juan, I. Ochandiano, L. Olivier, H. Álvarez, A. Herrero, C. Mantellini, M. Vicente, M. Sagué-Vilavella, L. Ilzarbe, L. Pintor, M. Garriga, V. Llorca-Bofí, M. Bioque

**Affiliations:** 1 Psychiatry and Psychology Department; 2 Bipolar and Depressive Disorders Unit; 3 Servei de Psiquiatria i Psicologia; 4 Consultation Liaison Psychiatry Unit; 5 Schizophrenia Unit, Hospital Clínic de Barcelona, Barcelona, Spain

## Abstract

**Introduction:**

Systemic lupus erythematosus (SLE) is an autoimmune disorder characterized by a wide range of symptoms, including neurological and psychiatric symptomatology. Between 37 and 95% of SLE patients present psychiatric symptoms, which can appear at any point in the disease and may present in various forms, ranging from more common conditions like depression and anxiety to rarer manifestations such as psychosis and catatonia (Carrión-Barberà *et al*. Autoimmunity Reviews 2021;20:102780).

**Objectives:**

To describe the case of a patient who presented a SLE debut with catatonic symptoms and to review neuropsychiatric and specifically catatonic symptoms in SLE patients.

**Methods:**

We present the case of a 32-year-old woman with no previous psychiatric history who was brought to our psychiatry emergency department (PED) for difficulties in language emission, only being able to babble for the last days. Approximately two weeks ago she had started to present anxiety, insomnia and catastrophic thoughts, so she consulted a psychiatrist who recommended starting escitalopram up to 15mg/d. In the next days she started to present difficulties of speech which worsened. Upon arrival, several diagnostic tests were performed including blood tests, CT scan, lumbar punction and electroencephalogram, showing no abnormalities. However, since the symptoms did not improve she was hospitalized for diagnostic affiliation.

**Results:**

During her hospitalization, more diagnostic tests were performed, including brain MRI and more exhaustive blood tests with autoimmunity profile, although the results were not available until several days later. Although the diagnosis was not yet clear and there were doubts among psychosis, neurologic functional disorder and organic mental disorder, several drugs were started including corticotherapy and olanzapine 15mg/d, venlafaxine up to 150mg/d and clonazepam 2.5mg/d with partial improvement of the symptoms, still showing motor retardation, blocking, perplexed gaze and mutism. In parallel, blood tests results emerged, showing high titre ANA antibodies, positive anti-double-stranded DNA and anti-Sm antibodies and decreased complement C3 component. With these findings, the diagnosis orientation was systemic lupus erythematosus (SLE).

**Conclusions:**

Catatonia is a rare psychomotor syndrome that can be associated with SLE. Given its rarity, there still no standardized treatment for it in SLE patients, although some options have been proposed including, as in any other SLE manifestations, flare immunosuppressive at high doses and in these patients, the usual psychiatric management (Boeke *et al*. Psychosomatics 2018;59:523-530; Sundaram *et al*. Rheumatology Journal 2021;42:1461-1476) Clinicians should keep this in mind to correctly diagnose and treat these patients, since early identification of this syndrome and prompt treatment leads to a more favorable outcome.

**Disclosure of Interest:**

None Declared

